# Unique Presentations of Invasive Lobular Breast Cancer: A Case Series

**Published:** 2014-12

**Authors:** Muhammad Tariq Shakoor, Samia Ayub, Ramesh Mohindra, Zunaira Ayub, Abdul Ahad

**Affiliations:** 1 Department of Internal Medicine, Baystate Medical Center (Tufts University School of Medicine), Springfield, MA, USA;; 2 Department of Pulmonary and Critical Care, University of Arkansas for Medical Sciences, Little Rock, AR, USA;; 3 Department of Hematology/Oncology, St Mary Mercy Hospital, Livonia, MI, USA;; 4 Medical Student, Fatima Jinnah Medical College, Lahore, Pakistan;; 5 Medical Student, Nishter Medical College, Multan, Pakistan

**Keywords:** Invasive lobular carcinoma (ILC), Invasive Ductal Carcinoma (IDC), Breast Cancer; metastasis

## Abstract

**Introduction:**

Breast carcinoma is the most common malignancy in women. Unlike IDC, which typically metastasizes to the lung, liver or bone, ILC has been found to metastasize to GI tract, peritoneum and retroperitoneum. Nonspecific symptomology may be considered secondary to other diseases and this can delay the definite diagnosis and treatment of metastatic disease. Knowledge of the pattern of disease spread is essential for accurate diagnosis and early initiation of systemic treatment, thus avoiding unnecessary interventions. We are reporting three unique cases of metastatic ILC presenting with wide range of symptoms.

**Case Presentations:**

Case A: 69-year-old female presented with recurrent jaundice. Case B: 77-year-old female with the past medical history of right breast ILC seven years ago status post right radical mastectomy with chemotherapy, presented with anemia. Case C: 56-year-old female presented with bright red blood per rectum.

**Conclusion:**

A high level of suspicion is needed for metastatic breast cancer in patients with history of ILC, regardless of disease free interval. Since it frequently metastasizes to unusual sites and presents with a wide spectrum of symptoms.

## INTRODUCTION

Breast carcinoma is the most common malignancy in women (1). Infiltrating ductal carcinoma is the most common histologic subtype of breast carcinoma and accounts for approximately 90% of all invasive breast cancers. Although lobular carcinoma accounts for only 10–14% of all breast cancers (2), given the high incidence of breast cancer in women; its incidence is greater than that of invasive cervical carcinoma and approximately two thirds that of ovarian cancer (3). With early diagnosis and treatment, many women can become long-term breast cancer survivors; however, recurrence and metastasis are quite common. The common metastatic sites for breast cancer are bone, lung, and liver in both ductal and lobular carcinomas. Invasive lobular carcinoma (ILC) has been found to frequently metastasize in gastrointestinal tract, peritoneum and retroperitoneum (4-7). Nonspecific symptomology may be considered secondary to other diseases and this can delay the definite diagnosis and treatment of metastatic disease. Knowledge of the pattern of disease spread is essential for accurate diagnosis and early initiation of systemic treatment, thus avoiding unnecessary interventions. We are reporting three unique cases of metastatic ILC presenting with wide range of symptoms.

## CASE PRESENTATIONS

### Case A: Invasive lobular carcinoma of the breast presenting as cholangiocarcinoma

**Figure 1:**
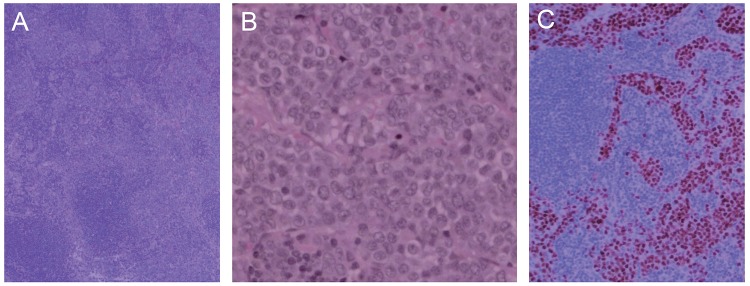
A) Lymph node containing deposits of metastatic lobular carcinoma expanding sinuses (hematoxylin and eosin, × 4); B) High-power view of neoplastic cells with little variation in nuclear contours (hematoxylin and eosin, × 40); C) Immunoperoxidase stain for estrogen receptor marking the nuclei of the malignant cells (10 ×).

A 69-year-old Caucasian female was referred to our hospital for evaluation of recurrent jaundice. Her past medical history consisted of cholecystitis status post cholecystectomy, squamous cell carcinoma of oral cavity status post-surgical resection, chemoradiotherapy and right-sided breast lobular cancer 30 years back status post mastectomy. Her initial labs showed hemoglobin (HGB) of 10.2 g/dl, platelet (PLT) 109000/μL, creatinine (Cr) 1.2 mg/dl (baseline Cr 1 mg/dl), aspartate aminotransferase (AST) 111 U/L, alanine aminotransferase (ALT) 166 U/L, total bilirubin 10.6 mg/dl, direct bilirubin 9.2 mg/dl, alkaline phosphatase (ALP) 513 U/L and lactate dehydrogenase (LDH) 130 U/L. US abdomen showed severe intra- and extra-hepatic biliary ductal dilatation with the common bile duct measuring up to 1.9 cm. Patient was admitted with the preliminary diagnosis of obstructive jaundice. Endoscopic retrograde cholangiopancreatography (ERCP) was done showing a stricture in the common bile duct. A temporary stent was placed after biopsy. Biopsy report showed adenocarcinoma of common bile duct, labeled as cholangiocarcinoma. Further imaging did not reveal any metastasis. Later on, we tried inserting a permanent stent as outpatient but failed secondary to unstable vital signs. Patient was readmitted in few months for fever associated with recurrent jaundice, with preliminary diagnosis of ascending cholangitis. Patient was started on appropriate intravenous antibiotics and was transferred to a tertiary care hospital for further management, as patient needed a permanent stent in common bile duct. In the tertiary care hospital gastroenterologist tried inserting a permanent stent but that was complicated with bile duct rupture. Emergent laparotomy was performed. Bile duct was repaired and the surgeon biopsied common bile duct and abdominal lymph nodes. Bile duct biopsy was negative but pericholedochal lymph nodes showed lobular breast cancer positive for estrogen-progesterone receptors but negative for Her-2 (Fig. 1A-1C).

### Case B: Invasive lobular carcinoma of the breast presenting with metastasis to stomach, retroperitoneum and bladder

**Figure 2:**
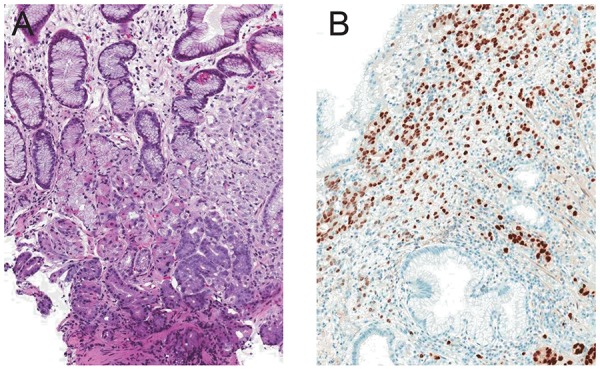
A) Gastric biopsy with metastatic lobular breast carcinoma replacing superficial lamina propria and infiltrating among normal gastric glands (arrow, hematoxylin and eosin, × 10); B) Immunoperoxidase stain for estrogen receptor marking the nuclei of the malignant cells (10 ×).

**Figure 3:**
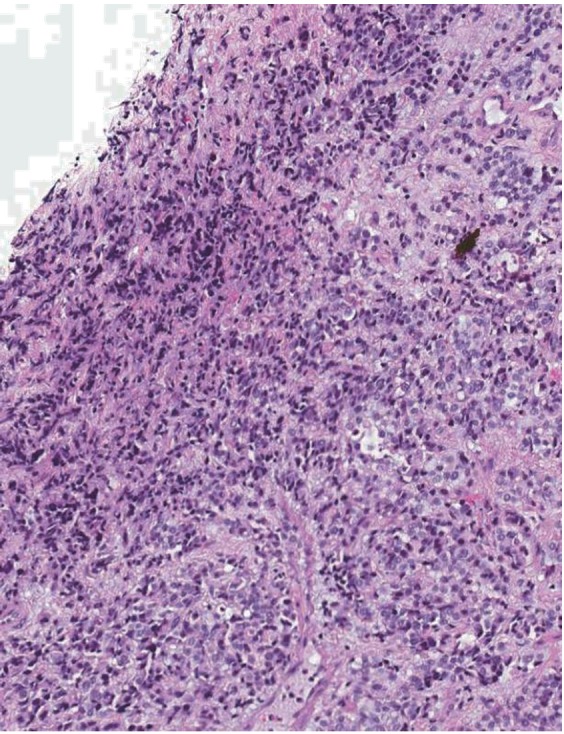
Bladder biopsy containing metastatic lobular breast carcinoma (hematoxylin and eosin, × 10).

A 77-year-old Caucasian female with the past medical history of right breast ILC (T2 N1 M0 stage 2B) seven years ago status post right radical mastectomy with chemotherapy (arimidex) admitted directly from her primary doctor's office for HGB of 7.8 g/dl (baseline HGB 12.5). Patient denied any shortness of breath, abdominal pain, hematemesis, rectal or vaginal bleeding, blood in stools, hematuria, recent weight loss, night sweats or fever. Her initial labs showed HGB of 7.8 g/dl, mean corpuscular volume (MCV) 92 fl, hematocrit (HCT) 23.6 %, mean cellular hemoglobin concentration (MCHC) 32.9 g/dl, red cell distribution width (RDW) 19 %, Glucose 171 mg/dl, ALP 106 U/L, LDH 211 U/L and albumin of 4.1, rest of the initial labs were within normal limits. One pack of red blood cells was transfused and patient was admitted for further work up. Further workup showed ferritin 444 ng/ml, TIBC 309 mcg/dl, Iron level 81 mcg/dl, haptoglobin 339 mg/dl, erythropoietin level 131 mlU/ml, c-reactive protein (CRP) < 0.2 mg/dl, absolute reticulocyte 95 B/L, reticulocyte percent 3.7%, Folic acid 17.80 ng/ml, vitamin B12 458 pg/ml and peripheral smear showed anisocytosis 1+, polychromasia 1+ and basophilic stippling 1+. Upper gastrointestinal endoscopy showed significant edema and erythema throughout the gastric body. Gastric body and antrum biopsies showed metastatic carcinoma, histomorphologic features and immunochemical profile was consistent with metastatic breast lobular carcinoma (Fig. 2A, Fig. 2B). Colonoscopy showed a sigmoidal polyp and histology was consistent with tubular adenoma. Extensive imaging was done as a part of metastatic work up. Computerized tomography (*CT*) abdomen with pelvis revealed bilateral hydronephrosis without any stone along the courses of the ureters and diffuse thickening of the stomach wall. Retroperitoneal ultrasound revealed marked bilateral hydronephroses with diffuse abnormal thickening of the bladder wall. Transuretheral resection of the bladder was done and biopsy report showed a high grade, poorly differentiate carcinoma. Morphology was compatible with a metastatic breast carcinoma further confirmed with immunohistostains (Fig. 3). Whole body positron emission tomography (*PET*) *scan* was done as a part of further workup and it revealed bilateral hydronephrosis, infiltrative haziness surrounding the left kidney and the retroperitoneum including both the ureters and marked asymmetric wall thickening of the urinary bladder. Initially the urologist tried retrograde insertion of a ureteral stent but could not do it because of tight strictures of ureters externally. Later, the urologist did percutaneous bilateral nephrostomy with retrograde insertion of ureteral stents. Patient was readmitted in intensive care unit with severe obstructive uropathy and septic shock secondary to urinary tract infection. Patient died on the second day of admission due to cardiac arrest.

### Case C: Invasive lobular carcinoma of the breast presenting as rectal bleeding:

**Figure 4:**
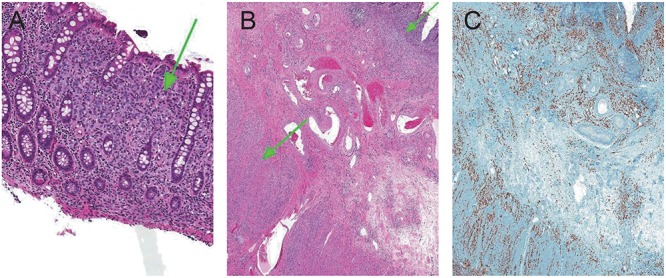
A) Colon biopsy with metastatic lobular breast carcinoma (arrow) replacing superficial lamina propria and infiltrating among normal colonic glands with regular tubular outlines (hematoxylin and eosin, × 10); B) Colon resection with full thickness involvement by metastatic lobular breast carcinoma (arrows, hematoxylin and eosin, × 2); C) Immunoperoxidase stain for estrogen receptor marking the nuclei of the malignant cells in the colon resection (2 ×).

**Figure 5:**
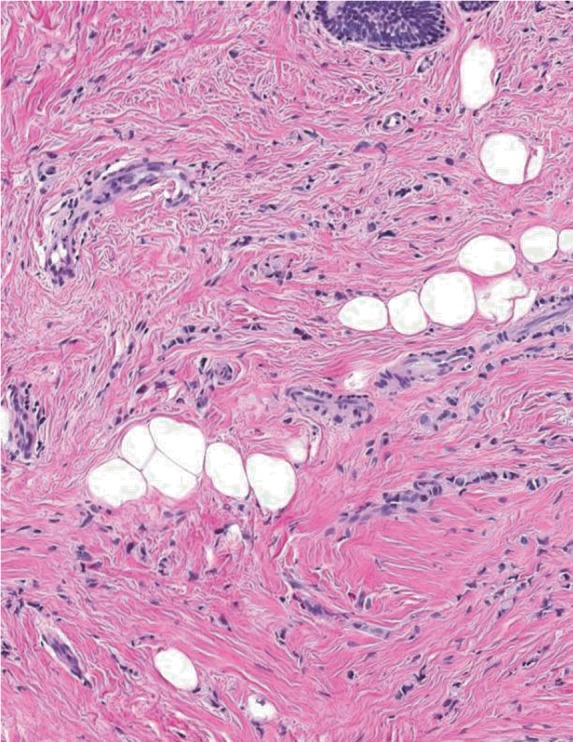
Cords and strands of infiltrative lobular breast carcinoma dissecting fibroadipose tissue of the breast and normal epithelium (hematoxylin and eosin, × 10).

A 56-year-old African American female with the past medical history of cholecystectomy presented with bright red blood per rectum for few hours. Patient did not complaint of any nausea, vomiting, abdominal pain, change in bowel movement, shortness of breath or palpitations. Digital rectal examination was normal. Her initial labs showed HGB 10.1 g/dl, HCT 29.9%, MCHC 33.9 g/dl, RDW 17.3%, PLT 64,000/μL, Cr 1.3 mg/dl and glucose 241 mg/dl, rest of the labs were within normal limits. Colonoscopy revealed small inflammatory area in cecum, suspicious for mild ischemia, with occasional diverticula but no polyp or mass was seen and random right colon biopsies showed diffuse metastatic adenocarcinoma. CT abdomen and pelvis with contrast showed diffuse, patchy hazy densities in omental fat and possible peritoneal metastasis. CT scan was also suspicious for bone metastasis but did not show any metastatic disease of any intra-abdominal organ. There was no evidence of bone metastasis on whole body bone scan. Carcinoembyonic antigen level was within normal limits. Later on during the hospital stay right colectomy was performed as the right colon was positive for metastatic adenocarcinoma. Intra-abdominal lymph nodes were biopsied. Patient tolerated the procedure really well and right colon was sent for histopathologic examination. Pathology report of the right colon revealed invasive poorly differentiated adenocarcinoma with signet ring cell, extensively involving lamina propria, submucosa, muscularis propria, serosa, mesenteric adipose tissue of colon appendix and small bowel. Proximal and distal surgical resection margins were positive for invasive carcinoma. All twenty-two regional lymph nodes were positive for metastatic carcinoma (Fig. 4A). Later on the patient followed oncologist in the office and on breast examination found a vague right upper quadrant mass mildly palpable. Bilateral digital screening mammography showed large areas of asymmetry with underlying calcification in right breast. Right breast diagnostic digital mammography and ultrasonography revealed extensive ill-defined asymmetry with in the right breast, essentially involving all four quadrants, with extensive suspicious calcification as well as a focal suspicious mass at 12 o’clock. Bone marrow biopsy was done because of consistent bicytopenia. A battery of immunohistochemical stain tests on resected colon, bone marrow and breast biopsy samples diagnosed estrogen-progesterone receptor positive and her-2 negative metastatic invasive lobular breast carcinoma (Fig. 4B, Fig. 4C). Patient was started on hormonal chemotherapy. Patient was readmitted, in one year, with epigastric pain and gastro duodenal endoscopy was done showing mild gastritis. Pathology report revealed diffuse foci of poorly differentiated adenocarcimoma in lamia propria, features consistent with metastatic lobular carcinoma originating from the breast. CT abdomen and pelvis showed significant retroperitoneal carcinomatosis. The whole retroperitoneum was infiltrated extensively with tumor. The rest of the colon and small bowel to mesentery were also infiltrated. Immunohistochemical stains revealed estrogen- progesterone receptor positive and her-2 negative metastatic invasive lobular breast carcinoma (Fig. 5).

## DISCUSSION

Breast cancer accounts for 19% of cancer deaths in women (8). Current therapies have had a significant impact on the mortality and survival rates of breast cancer (9). Prognosis is related to several factors including the presence of metastases and tumor histology being one of the predictors of metastatic spread. Some 30-80% of patients will develop metastatic disease following therapy. Jain *et al* (10) examined 1238 patients with breast cancer and identified the pattern of metastatic disease. They found that infiltrating ductal carcinoma (IDC) recurred more commonly in lung, pleura and bone, while ILC metastasized more to the bone marrow (*P*<0.01) and peritoneum (*P*<0.01). Bone involvement as the initial presentation of distant metastatic disease occurred in over 50% of the women with ILC, significantly more often than in those with IDC (34%). But the survival was similar for the two groups. In comparing the metastatic patterns of ILC versus IDC, Borst *et al* (11) found that ILC was significantly more likely to spread to the GI tract, gynecologic organs, peritoneal surface and retroperitoneum. This behavior, as explained by various studies, is due to the small size and shape of ILC cells, with E-cadherin overexpression favoring dis-cohesiveness between the cells to migrate to areas of microanatomy more conducive to stopping these cells (12, 13). Alternatively, the microenvironment of the ovary or peritoneum may provide growth and survival factors that favor ILC cells over IDC cells, explaining the difference in the metastatic pattern between the two subtypes (14).

Late development of metastatic disease in unique anatomical locations is a known characteristic of ILC. The maximum time from diagnosis of the primitive tumor to metastases was reported in a case of gastric metastasis from breast cancer occurred 30 years after surgery for primary tumor like in case A (15). The primitive tumor to metastasis interval of 30 years in our case A is one of the longest in the literature. Our case A patient presented with isolated symptom of recurrent jaundice and occult ILC metastatic site (common bile duct). Isolated metastatic symptom as an initial sign of invasive breast carcinoma without any other metastatic spread is very rare (16). Gastrointestinal metastasis from breast carcinoma may mimic a primary bowel tumor or other gastrointestinal disorder. Like initially we were thinking that it is a primary common bile duct tumor, especially because of isolated metastasis. Although rare, instances of occult ILC presenting initially with GI disease have been reported (11, 17). In an extensive review of the literature Massimo et al found 206 patients reported with GI tract involvement from breast cancer, from 1943 to June 2012 (18). According to the researchers each part of the GI tract has been reported involved, from tongue to the anus but it did not include the hepatobiliary system. In literature there is only one case report of breast cancer metastasis to biliary system (19). Our case A is second case report in history reporting isolated biliary system metastasis.

We could not find any case report in literature reporting ILC metastasis to the urinary bladder. To the best of our knowledge our case B is the first case report in literature reporting ILC metastasis to urinary bladder. Cases of ILC metastasizing to the peritoneal cavity and retroperitoneum are not unusual. Ureteral obstruction is a common presentation with retroperitoneal involvement (20). However, sometimes the metastatic ILC presents with isolated retroperitoneal fibrosis (RPF) (21) and it is very difficult to diagnose RPF as metastatic ILC on clinical/ radiologic grounds, a high degree of suspicion of a metastatic neoplasm should be maintained in cases of isolated RPF. It is always necessary to exclude metastatic carcinoma as an initiating cause, before diagnosing idiopathic RPF. In our case B, patient presented with vast metastasis to gut, peritoneum, retroperitonium, urinary bladder and bone marrow.

Our case C presented with rectal bleeding caused by metastasis from an undiagnosed breast cancer. Simultaneous breast cancer and intestinal obstruction have been described in few reports (21-23), but rectal bleeding with an undiagnosed breast cancer has never been described. Our third case also highlights the importance of breast cancer screening as patient was never screened for a breast cancer. Although rare, instances of occult ILC presenting initially with GI disease have been reported (24). Case reports of GI metastases as an initial presentation of ILC include lesions in the stomach, rectum, peritoneum, and/or retroperitoneum (25-29).

It is very difficult to distinguish primary from metastatic tumors, features that are typically used to make this distinction include the gross configuration of the tumor (17) and a detailed immunohistochemical analysis. Metastatic breast cancers are usually positive for CK7, CEA, ER, PR and GCDFP-15. CK7 and CEA positivity is nonspecific. CK20, on the other hand, is almost always present in gastrointestinal tumors and absent in breast carcinomas (30). Clinicians involved in the management of this disease should also be aware of the limitations of endoscopic biopsies in excluding metastasis because of a high false negative rate from the relatively late involvement of the avascular mucosal layers with metastasis. Therefore, non-conventional techniques, like macro-biopsies or endoscopic ultrasound guided fine needle aspiration cytology, should be employed in cases where there is a high index of clinical suspicion (31). The therapeutic approach to these patients is still object of debate. Surgery is mainly indicated to solve the complications but it does not extend the patient survival, systemic hormonal therapy or chemotherapy is commonly the main treatment (32).

## CONCLUSION

Unlike IDC, which typically metastasizes to the lung, liver or bone, ILC has been found to metastasize to GI tract, peritoneum and retroperitoneum. Our primary aim was to illustrate these situations. We presented a unique case of metastatic ILC with isolated metastasis to common bile duct after 30 years of disease free interval. One and only case of ILC with metastasis to urinary bladder, stomach and retroperitonium and a rare case of metastatic ILC, initially presenting with rectal bleed later diagnosed as metastatic breast cancer, with an undiagnosed primary.

Due to the unique characteristics of ILC, we propose a closer follow up with adequate diagnostic procedures, like CT scan, PET scan and endoscopy if suspicion, even if prolonged disease free interval. A high level of suspicion is needed for metastatic breast cancer, especially in patients with history of ILC as it frequently metastasizes to unusual sites and presents with wide spectrum of symptoms.

To the best of our knowledge, in literature this is the first case report of urinary bladder metastasis from breast cancer, second case report of a solitary common bile duct metastasis from breast carcinoma presenting as recurrent jaundice and second case report reporting 30 years of disease free interval from breast cancer diagnosis to metastasis.

## References

[1] Henderson CL, Henderson GP, Lawrence W, Lerhard RE (1995). Breast cancer. American Cancer Society textbook of clinical oncology.

[2] Rosen PP, Rosen PP (1997). Invasive lobular carcinoma. Rosen’s breast pathology.

[3] Parker SL, Tong L, Bolden S, Wingo PA (1997). Cancer statistics 1997. CA: A Cancer Journal for Clinicians January/February.

[1] Borst MJ (1993). Ingold JA: Metastatic patterns of invasive lobular versus invasive ductal carcinoma of the breast. Surgery.

[5] Harris M, Howell A, Chrissohou M, Swindell RI (1984). A comparison of the metastatic pattern of infiltrating lobular carcinoma and infiltrating duct carcinoma of the breast. Br. J. Cancer.

[6] Lamovec J, Bracko M (1991). Metastatic pattern of infiltrating lobular carcinoma of the breast: an autopsy study. J. Surg. Oncol.

[7] Winston CB, Hadar O, Teitcher JB, Caravelli JF (2000). Metastatic lobular carcinoma of the breast: patterns of spread in the chest, abdomen, and pelvis on CT. Am. J. Roentgenol.

[8] Rubin P (1993). Clinical Oncology. WB Saunders Co.

[9] Chu KC, Tarone RE, Kessler LG, Ries LA (1996). Recent trends in U.S. breast cancer incidence, survival, and mortality rates. J. Natl. Cancer Inst.

[10] Jain S, Fisher C, Smith P, Millis RR (1993). Patterns of metastatic breast cancer in relation to histological type. Eur. J. Cancer.

[11] Borst MJ, Ingold JA (1993). Metastatic patterns of invasive lobular versus invasive ductal carcinoma of the breast. Surgery.

[12] Lehr HA, Folpe A, Yaziji H, Kommoss F (2000). Cytokeratin 8 immunostaining pattern and E-cadherin expression distinguish lobular from ductal breast carcinoma. Am. J. Clin. Pathol.

[13] Sastre-Garau X, Jouve M, Asselain B, Vincent-Salomon A (1996). Infiltrating lobular carcinoma of the breast. Clinicopathologic analysis of 975 cases with reference to data on conservative therapy and metastatic patterns. Cancer.

[14] Arpino G, Bardou VJ, Clark GM, Elledge RM (2004). Infiltrating lobular carcinoma of the breast: tumor characteristics and clinical outcome. Breast Cancer Res.

[15] Benfiguig A, Anciaux ML, Eugene C, Benkemoun G (1992). Gastric metastasis of a cancer of the breast occurring after a cancer-free interval of 30 years. Ann. Gastroenterol Hepatol. (Paris).

[16] Hsieh PS, Yeh CY, Chen JR, Changchien CR (2004). Ileocecal breast carcinoma metastasis. Int. J. Colorectal Dis.

[17] Nazareno J, Taves D, Preiksaitis HG (2006). Metastatic breast cancer to the gastrointestinal tract: a case series and review of the literature. World J. Gastroenterol.

[18] Massimo A (2012). Metastatic Breast Cancer to the Gastrointestinal Tract:Report of Five Cases and Review of the Literature. International Journal of Breast Cancer.

[19] Franco D, Martin B, Smadja C, Szekely AM (1987). Biliary metastases of breast carcinoma. The case for resection. Cancer.

[20] Sironi M (2011). Rare abdominal metastases from occult lobular breast cancer:report of two cases. Updates Surg.

[21] Yousaf GM (2010). Invasive lobular carcinoma of the breast presenting as retroperitoneal fibrosis: a case report. Journal of Medical Case Reports.

[22] Calo PG, Fanni D, Ionta MT, Medas F (2012). Jejunal obstruction caused by metastasis from an undiagnosed breast cancer: a case report. Tumori.

[23] Kobayashi T, Adachi S, Matsuda Y, Tominaga S (2007). A case of metastatic lobular breast carcinoma with detection of the primary tumor after ten years. Breast Cancer.

[24] Lottini M (2002). Duodenal obstruction from isolated breast cancer metastasis: a case report. Tumori.

[25] Mistrangelo M (2011). Obstructive colon metastases from lobular breast cancer: report of a case and review of the literature. Tumori.

[26] Nazareno J, Taves D, Preiksaitis HG (2006). Metastatic breast cancer to the gastrointestinal tract: a case series and review of the literature. World J. Gastroenterol.

[27] Neal L, Sookhan N, Reynolds C (2009). Occult breast carcinoma presenting as gastrointestinal metastases. Case Report Med.

[28] Arrangoiz R (2011). Case report and literature review: Metastatic lobular carcinoma of the breast an unusual presentation. Int. J. Surg. Case Rep.

[29] Clavien PA, Laffer U, Torhost J (1990). Gastro-intestinal metastases as first clinical manifestation of the dissemination of a breast cancer. Eur. J. Surg. Oncol.

[30] Estrella JS, Wu TT, Rashid A, Abraham SC (2011). Mucosal colonization by metastatic carcinoma in the gastrointestinal tract: a potential mimic of primary neoplasia. Am. J. Surg. Pathol.

[31] Almubarak MM (2011). Gastric metastasis of breast cancer: a single centre retrospective study. Dig. Liver Dis.

[32] Théraux J, Bretagnol F, Guedj N, Cazals-Hatem D (2009). Colorectal breast carcinoma metastasis diagnosed as an obstructive colonic primary tumor. A case report and review of the literature. Gastroenterol Clin. Biol.

